# 5-Chloro­methyl-1,3-dimethyl-1*H*-pyrazole

**DOI:** 10.1107/S1600536810042844

**Published:** 2010-10-31

**Authors:** Guiqiu Yang, Hongcai Xu, Huibin Yang, Haibo Yu

**Affiliations:** a Shenyang University of Chemical Technology, Shenyang 110142, People’s Republic of China; b Pharmaceutical Division, Shenyang University of Chemical Technology, Shenyang 110142, People’s Republic of China; c Agrochemicals Division, Shenyang Research Institute of Chemical Industry, Shenyang 110021, People’s Republic of China

## Abstract

The pyazole ring in the title compound, C_6_H_9_ClN_2_, is almost planar (r.m.s. deviation = 0.003 Å). In the crystal, mol­ecules are linked by C—H⋯N inter­actions, forming [100] chains.

## Related literature

For a related structure, see: Baldy *et al.* (1985 ▶).
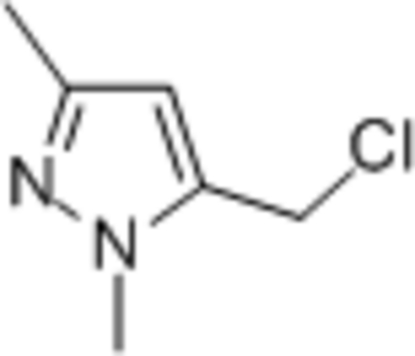


## Experimental

### 

#### Crystal data

C_6_H_9_ClN_2_
*M* *_r_* = 144.60
Triclinic, 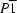

*a* = 6.5210 (7) Å
*b* = 7.3111 (7) Å
*c* = 7.9854 (8) Å
α = 88.383 (1)°
β = 77.563 (2)°
γ = 85.725 (2)°
*V* = 370.71 (6) Å^3^
*Z* = 2
Mo *K*α radiation
μ = 0.43 mm^−1^
*T* = 296 K
0.28 × 0.22 × 0.20 mm

#### Data collection

Bruker SMART CCD diffractometer
Absorption correction: multi-scan (*SADABS*; Bruker, 2001 ▶) *T*_min_ = 0.890, *T*_max_ = 0.919
1906 measured reflections
1304 independent reflections
1135 reflections with *I* > 2σ(*I*)
*R*_int_ = 0.011

#### Refinement

*R*[*F* ^2^ > 2σ(*F* ^2^)] = 0.038
*wR*(*F* ^2^) = 0.105
*S* = 1.05
1304 reflections
85 parameters
H-atom parameters constrained
Δρ_max_ = 0.22 e Å^−3^
Δρ_min_ = −0.31 e Å^−3^

### 

Data collection: *SMART* (Bruker, 2001 ▶); cell refinement: *SAINT* (Bruker, 2001 ▶); data reduction: *SAINT*; program(s) used to solve structure: *SHELXS97* (Sheldrick, 2008 ▶); program(s) used to refine structure: *SHELXL97* (Sheldrick, 2008 ▶); molecular graphics: *SHELXTL* (Sheldrick, 2008 ▶); software used to prepare material for publication: *SHELXTL*.

## Supplementary Material

Crystal structure: contains datablocks I, global. DOI: 10.1107/S1600536810042844/hb5664sup1.cif

Structure factors: contains datablocks I. DOI: 10.1107/S1600536810042844/hb5664Isup2.hkl

Additional supplementary materials:  crystallographic information; 3D view; checkCIF report

## Figures and Tables

**Table 1:** Hydrogen-bond geometry (Å, °)

*D*—H⋯*A*	*D*—H	H⋯*A*	*D*⋯*A*	*D*—H⋯*A*
C1—H1*B*⋯N2^i^	0.97	2.50	3.446 (3)	164

Symmetry code: (i) 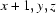.

**Fig. 1.**
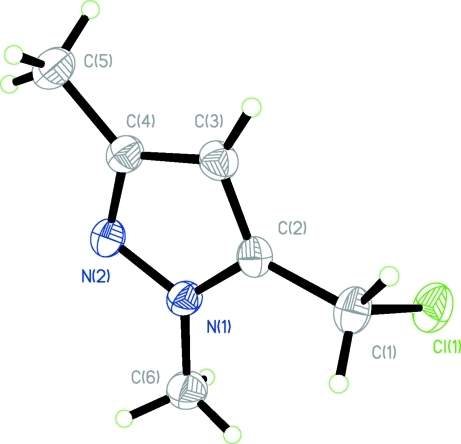
The molecular structure of (I), with 30% probability displacement ellipsoids.

## supplementary crystallographic information

### Experimental

*N*,*N*-Dimethylformamide(0.96 g, 11 mmol) was add to a 100 ml three necked-bottle, and phosphoryl trichloride(6.13 g, 40 mmol) was added slowly under ice-bath, then added (1,3-dimethyl-1*H*-pyrazole-5-yl)methanol(1.26 g, 10 mmol) at room temperature in portions. The reaction mixture was heated to reflux and reacted for 6 h. After separation through silica gel column chromatography (fluent: ethyl acetate/petroleum ether=1/5). The title product compound was obtained as a white solid (0.36 g, 22%) and recrystallised from methylene chloride to yield colourless blocks of (I).

Anal. Calcd for C_6_H_9_N_2_: C, 49.84; H, 6.27; N, 19.37. Found: C, 49.81; H, 6.30; N, 19.40. ^1^H NMR(CDCl3): 2.22(s,3*H*, CH_3_), 3.84 (s,3*H*, N—CH_3_), 4.53(s, 2H, CH_2_), 6.04 (s, 1H, Pyrazole-H).

### Refinement

Although all H atoms were visible in difference maps, they werefinally placed in geometrically calculated positions, with C-Hdistances in the range 0.93–0.96Å and N—H distances of 0.86 Å, andincluded in the final refinement in the riding model approximation,with *U*_iso_(H) = 1.2*U*_eq_(C, N) and *U*_iso_(H) = 1.5*U*_eq_(C).

### Crystal data

**Table:**

C_6_H_9_ClN_2_	*Z* = 2
*M**_r_* = 144.60	*F*(000) = 152
Triclinic, *P*1	*D*_x_ = 1.295 Mg m^−^^3^
Hall symbol: -P 1	Melting point = 361–364 K
*a* = 6.5210 (7) Å	Mo *K*α radiation, λ = 0.71073 Å
*b* = 7.3111 (7) Å	Cell parameters from 1109 reflections
*c* = 7.9854 (8) Å	θ = 2.6–26.7°
α = 88.383 (1)°	µ = 0.43 mm^−^^1^
β = 77.563 (2)°	*T* = 296 K
γ = 85.725 (2)°	BLOCK, colorless
*V* = 370.71 (6) Å^3^	0.28 × 0.22 × 0.20 mm

### Data collection

**Table:**

Bruker SMART CCD diffractometer	1304 independent reflections
Radiation source: fine-focus sealed tube	1135 reflections with *I* > 2σ(*I*)
graphite	*R*_int_ = 0.011
φ and ω scans	θ_max_ = 25.0°, θ_min_ = 2.6°
Absorption correction: multi-scan (*SADABS*; Bruker, 2001)	*h* = −7→4
*T*_min_ = 0.890, *T*_max_ = 0.919	*k* = −7→8
1906 measured reflections	*l* = −9→8

### Refinement

**Table:**

Refinement on *F*^2^	Secondary atom site location: difference Fourier map
Least-squares matrix: full	Hydrogen site location: inferred from neighbouring sites
*R*[*F*^2^ > 2σ(*F*^2^)] = 0.038	H-atom parameters constrained
*wR*(*F*^2^) = 0.105	*w* = 1/[σ^2^(*F*_o_^2^) + (0.0513*P*)^2^ + 0.1537*P*] where *P* = (*F*_o_^2^ + 2*F*_c_^2^)/3
*S* = 1.05	(Δ/σ)_max_ < 0.001
1304 reflections	Δρ_max_ = 0.22 e Å^−^^3^
85 parameters	Δρ_min_ = −0.30 e Å^−^^3^
0 restraints	Extinction correction: *SHELXL97* (Sheldrick, 2008), Fc^*^=kFc[1+0.001xFc^2^λ^3^/sin(2θ)]^-1/4^
Primary atom site location: structure-invariant direct methods	Extinction coefficient: 0.43 (3)

### Special details

**Table:**

Geometry. All e.s.d.'s (except the e.s.d. in the dihedral angle between two l.s. planes) are estimated using the full covariance matrix. The cell e.s.d.'s are taken into account individually in the estimation of e.s.d.'s in distances, angles and torsion angles; correlations between e.s.d.'s in cell parameters are only used when they are defined by crystal symmetry. An approximate (isotropic) treatment of cell e.s.d.'s is used for estimating e.s.d.'s involving l.s. planes.
Refinement. Refinement of *F*^2^ against ALL reflections. The weighted *R*-factor *wR* and goodness of fit *S* are based on *F*^2^, conventional *R*-factors *R* are based on *F*, with *F* set to zero for negative *F*^2^. The threshold expression of *F*^2^ > σ(*F*^2^) is used only for calculating *R*-factors(gt) *etc*. and is not relevant to the choice of reflections for refinement. *R*-factors based on *F*^2^ are statistically about twice as large as those based on *F*, and *R*- factors based on ALL data will be even larger.

### Fractional atomic coordinates and isotropic or equivalent isotropic displacement parameters (Å^2^)

**Table:**

	*x*	*y*	*z*	*U*_iso_*/*U*_eq_	
Cl1	1.05348 (10)	0.89562 (8)	0.28229 (8)	0.0675 (3)	
N1	0.7203 (2)	0.6178 (2)	0.1574 (2)	0.0414 (4)	
N2	0.5716 (3)	0.4983 (2)	0.2188 (2)	0.0450 (4)	
C1	1.0872 (3)	0.6828 (3)	0.1664 (3)	0.0509 (5)	
H1A	1.1141	0.7093	0.0443	0.061*	
H1B	1.2085	0.6102	0.1904	0.061*	
C2	0.8994 (3)	0.5757 (3)	0.2144 (2)	0.0408 (5)	
C3	0.8653 (3)	0.4224 (3)	0.3159 (3)	0.0453 (5)	
H3	0.9593	0.3600	0.3737	0.054*	
C4	0.6600 (3)	0.3784 (3)	0.3150 (2)	0.0435 (5)	
C5	0.5411 (4)	0.2246 (3)	0.4043 (3)	0.0586 (6)	
H5A	0.3958	0.2440	0.3976	0.088*	
H5B	0.5518	0.2198	0.5224	0.088*	
H5C	0.5991	0.1110	0.3504	0.088*	
C6	0.6713 (4)	0.7699 (3)	0.0489 (3)	0.0579 (6)	
H6A	0.5882	0.8648	0.1187	0.087*	
H6B	0.5937	0.7284	−0.0304	0.087*	
H6C	0.7997	0.8172	−0.0134	0.087*	

### Atomic displacement parameters (Å^2^)

**Table:**

	*U*^11^	*U*^22^	*U*^33^	*U*^12^	*U*^13^	*U*^23^
Cl1	0.0728 (5)	0.0529 (4)	0.0782 (5)	−0.0180 (3)	−0.0135 (3)	−0.0100 (3)
N1	0.0373 (9)	0.0436 (9)	0.0435 (9)	−0.0058 (7)	−0.0085 (7)	0.0046 (7)
N2	0.0381 (9)	0.0480 (10)	0.0493 (9)	−0.0092 (7)	−0.0085 (7)	0.0007 (7)
C1	0.0387 (11)	0.0513 (12)	0.0616 (13)	−0.0059 (9)	−0.0065 (9)	−0.0068 (10)
C2	0.0346 (10)	0.0425 (10)	0.0449 (10)	−0.0009 (8)	−0.0073 (8)	−0.0066 (8)
C3	0.0455 (11)	0.0434 (11)	0.0487 (11)	0.0015 (9)	−0.0157 (9)	−0.0001 (8)
C4	0.0467 (11)	0.0402 (10)	0.0417 (10)	−0.0053 (8)	−0.0044 (8)	−0.0028 (8)
C5	0.0652 (15)	0.0492 (12)	0.0589 (13)	−0.0133 (11)	−0.0054 (11)	0.0036 (10)
C6	0.0584 (14)	0.0556 (13)	0.0635 (14)	−0.0074 (11)	−0.0219 (11)	0.0140 (11)

### Geometric parameters (Å, °)

**Table:**

Cl1—C1	1.808 (2)	C3—C4	1.401 (3)
N1—C2	1.354 (2)	C3—H3	0.9300
N1—N2	1.357 (2)	C4—C5	1.490 (3)
N1—C6	1.449 (3)	C5—H5A	0.9600
N2—C4	1.330 (3)	C5—H5B	0.9600
C1—C2	1.478 (3)	C5—H5C	0.9600
C1—H1A	0.9700	C6—H6A	0.9600
C1—H1B	0.9700	C6—H6B	0.9600
C2—C3	1.368 (3)	C6—H6C	0.9600
			
C2—N1—N2	111.77 (16)	N2—C4—C3	110.45 (17)
C2—N1—C6	128.92 (17)	N2—C4—C5	120.51 (19)
N2—N1—C6	119.29 (16)	C3—C4—C5	129.03 (19)
C4—N2—N1	105.36 (15)	C4—C5—H5A	109.5
C2—C1—Cl1	111.73 (14)	C4—C5—H5B	109.5
C2—C1—H1A	109.3	H5A—C5—H5B	109.5
Cl1—C1—H1A	109.3	C4—C5—H5C	109.5
C2—C1—H1B	109.3	H5A—C5—H5C	109.5
Cl1—C1—H1B	109.3	H5B—C5—H5C	109.5
H1A—C1—H1B	107.9	N1—C6—H6A	109.5
N1—C2—C3	106.40 (17)	N1—C6—H6B	109.5
N1—C2—C1	123.08 (18)	H6A—C6—H6B	109.5
C3—C2—C1	130.52 (19)	N1—C6—H6C	109.5
C2—C3—C4	106.02 (17)	H6A—C6—H6C	109.5
C2—C3—H3	127.0	H6B—C6—H6C	109.5
C4—C3—H3	127.0		
			
C2—N1—N2—C4	−0.5 (2)	Cl1—C1—C2—C3	−104.4 (2)
C6—N1—N2—C4	−178.81 (18)	N1—C2—C3—C4	−0.1 (2)
N2—N1—C2—C3	0.3 (2)	C1—C2—C3—C4	−179.3 (2)
C6—N1—C2—C3	178.48 (19)	N1—N2—C4—C3	0.4 (2)
N2—N1—C2—C1	179.62 (17)	N1—N2—C4—C5	−179.94 (17)
C6—N1—C2—C1	−2.2 (3)	C2—C3—C4—N2	−0.2 (2)
Cl1—C1—C2—N1	76.5 (2)	C2—C3—C4—C5	−179.8 (2)

### Hydrogen-bond geometry (Å, °)

**Table:**

*D*—H···*A*	*D*—H	H···*A*	*D*···*A*	*D*—H···*A*
C1—H1B···N2^i^	0.97	2.50	3.446 (3)	164

Symmetry codes: (i) *x*+1, *y*, *z*.
